# Connecting the Dots: Interplay of Pathogenic Mechanisms between COVID-19 Disease and Mucormycosis

**DOI:** 10.3390/jof7080616

**Published:** 2021-07-29

**Authors:** Hariprasath Prakash, Anna Skiada, Raees Ahmad Paul, Arunaloke Chakrabarti, Shivaprakash Mandya Rudramurthy

**Affiliations:** 1 Medical Microbiology, Department of Public Health, International Higher School of Medicine, Issyk-Kul Regional Campus, Cholpon-Ata 722125, Kyrgyzstan; harisath2003@gmail.com; 2 First Department of Medicine, Laiko Hospital, National and Kapodistrian University of Athens, 11527 Athens, Greece; askiada@yahoo.com; 3 Department of Medical Microbiology, Postgraduate Institute of Medical Education and Research, Chandigarh 160012, India; raeesbio@gmail.com (R.A.P.); arunaloke@hotmail.com (A.C.)

**Keywords:** COVID-19, mucormycosis, Mucorales, GRP78, EGFR, iron, corticosteroid therapy, diabetes mellitus

## Abstract

Coronavirus disease (COVID-19)-associated mucormycosis (CAM) is an emerging threat globally, especially in India. More than 40,000 CAM cases have been reported in India. The emergence of CAM cases in India has been attributed to environmental, host, and iatrogenic factors. Mucorales spore burden has been reported globally; however, their presence is higher in tropical countries such as India, contributing to the emergence of CAM. Before the COVID-19 pandemic, patients with diabetes mellitus, haematological malignancies, solid organ transplants, corticosteroid therapy and neutropenia were more prone to mucormycosis, whereas in COVID-19 patients, virus-induced endothelial dysfunction, hyperglycaemia, and immune dysfunction following corticosteroid use increase the risk of acquiring mucormycosis. The interaction of Mucorales spores with the epithelial cells, followed by endothelial invasion, is a crucial step in the pathogenesis of mucormycosis. Endothelial damage and increased endothelial receptor expression induced by COVID-19 infection may predispose patients to CAM. COVID-19 infection may directly induce hyperglycaemia by damaging beta cells of the pancreas or by corticosteroid therapy, which may contribute to CAM pathogenesis. Iron acquisition from the host, especially in diabetic ketoacidosis (DKA) or deferoxamine therapy, is an important virulence trait of Mucorales. Similarly, the hyperferritinaemia caused by COVID-19 may act as a source of iron for Mucorales growth and invasion. In addition, corticosteroid treatment reduces or abolishes the innate immune functions of phagocytic cells contributing to the pathogenesis of CAM. This review aims to discuss primarily the host and iatrogenic factors shared between COVID-19 and mucormycosis that could explain the emergence of CAM.

## 1. Introduction

Mucormycosis is an angio-invasive infection characterised by tissue necrosis and infarction of the blood vessels [1]. Mucormycosis is caused by saprophytic fungi that belong to the order Mucorales [2,3]. Compared to global data, the case burden of mucormycosis was estimated to be 70 times higher in India before the COVID-19 pandemic emerged [4,5]. A multi-country study on COVID-19-associated mucormycosis (CAM) reported that 53% of the cases are from India, followed by the United States of America (10%), Pakistan (6.3%), France (5%), Mexico (5%), Iran (5%), and Russia (2.5%) [6]. During this pandemic, the caseload of mucormycosis increased overwhelmingly (>40,000 cases) in India, prompting the Indian health authorities to declare mucormycosis as a notifiable disease [7]. The situation is aggravated due to the limited availability of first-line antifungal drugs, such as liposomal amphotericin or amphotericin B deoxycholate. Considering the present crisis, the European Confederation of Medical Mycology (ECMM) and the International Society for Human and Animal Mycology (ISHAM) have proposed guidelines for the management of mucormycosis in low and middle-income countries [7]; further, they have also proposed global guidelines for managing mucormycosis [8]. Before the COVID-19 pandemic, diabetes mellitus was reported as the most common risk factor for mucormycosis in India, followed by haematological malignancies and solid organ transplant recipients [4,5]. Mucormycosis has also been reported in patients with no underlying illness [4,5]. A multicentre study in India had identified diabetes, inappropriate steroid therapy (6 mg of dexamethasone per day for 7 to 10 days is recommended; a higher dose and a longer treatment duration are considered to be inappropriate), and the COVID-19 virus as risk factors for the increase in mucormycosis cases during the first wave of COVID-19 in 2020 [9]. During this outbreak, rhino-orbital-cerebral mucormycosis (ROCM) was the most common presentation, followed by pulmonary mucormycosis [6,9].

An environmental study on the ecology of Mucorales in Indian soils reported a high prevalence of clinically relevant Mucorales [2]. Mucorales spores are also highly prevalent in the indoor and outdoor air of the same country [10]. Patients acquire the infection by inhalation, ingestion or traumatic inoculation of the spores from the environment. The reasons for this CAM outbreak could be multifactorial. Other than environmental factors, uncontrolled diabetes mellitus, inappropriate steroid therapy, increased iron accumulation, and the damage caused by the COVID-19 virus may be driving this outbreak [9,11,12,13]. This review attempts to elucidate the interplay of risk factors or possible pathogenic mechanisms in the emergence of CAM.

## 2. Environmental Factors and Mucorales: A Possible Reason for the Surge in CAM

Humans acquire mucormycosis by inhalation, ingestion or traumatic inoculation of the sporangiospores of Mucorales from the environment [2,3]. Mucorales have ubiquitous distribution; however, the spore burden is higher in tropical countries [2,14]. Mucorales spores have been isolated from air in India′s indoor and outdoor environment [10,14]. *Rhizopus arrhizus,* the major pathogenic species, is also the predominant species isolated from the environment [2,4,14,15]. The number of CAM cases is very high in India compared to the rest of the world during this COVID-19 pandemic [6,11], and this high number may be linked to the high spore burden of pathogenic Mucorales in the environment. A study reported the isolation of rare species such as *Apophysomyces variabilis* and *Rhizopus homothallicus* in the Indian environment [2]. Infections due to these rare species are also prevalent in India [4,5]. *Rhizopus homothallicus* has been isolated from clinical samples at multiple centres even during the present outbreak besides the common species, *R. arrhizus* (unpublished data). A systematic environmental study is warranted during this outbreak; further comparing the environmental and clinical isolates may provide a plausible explanation for CAM emergence. The high case burden of CAM in India may also be linked to the emergence of virulence strains in the order Mucorales. Genome analysis of Mucorales isolated during the pre-COVID-19 and pandemic period, along with in vivo animal experiments, may bring out the possible role of virulence factors of Mucorales in the emergence of CAM.

## 3. Host and Iatrogenic Factors in the Pathogenesis of CAM

Host factors are likely to have a greater role in the increased case burden of CAM. Patients with diabetes mellitus and haematological malignancy and transplant recipients were at high risk of acquiring mucormycosis in the pre-COVID-19 era [5,16,17]. In comparison, patients with diabetes mellitus and inappropriately high doses of corticosteroid use are at increased risk of acquiring mucormycosis in the COVID-19 pandemic [6,9,11,18]. Hyperglycaemia in COVID-19 patients may be due to four reasons: (a) pre-existing diabetes mellitus, (b) damage of the beta cell pancreas by COVID-19 leading to the diminution of insulin production [19,20], (c) corticosteroid therapy [21,22], and (d) stress-related increased cortisol levels [23,24]. Hyperglycaemia and glucocorticoid treatment impair phagocytic functions, failing to arrest spore germination and growth and leading to disease progression [25,26]. In addition, patients with diabetes mellitus and COVID-19 exhibit increased ferritin levels (hyperferritinaemia), leading to altered iron homeostasis [27,28,29]. Further, endothelial damage and increased expression of endothelial receptors have been seen in COVID-19 patients [30,31,32]. Thus, the factors mentioned above, such as pre-existing endothelial damage and up-regulated endothelial receptors, glucocorticoid therapy, hyperglycaemia-associated complications such as hyperferritinaemia, and immune dysfunction of innate immune cells, are likely to contribute to the pathogenesis of CAM.

### 3.1. Endothelial Cells and Their Receptors: An Interface in COVID-19 Disease and Mucormycosis

**Figure 1:**
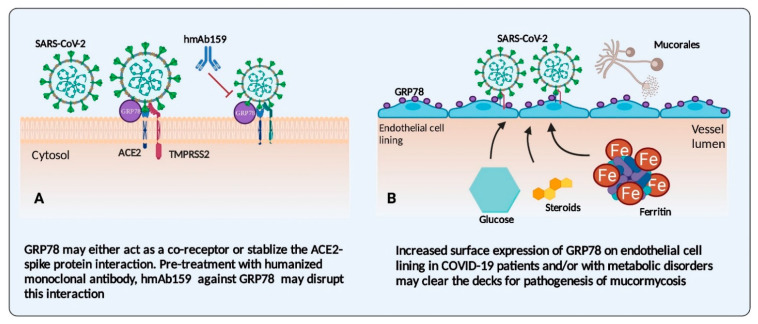
Glucose regulated protein (GRP78) in the pathogenesis of COVID-19-associated mucormycosis. (**A**) Angiotensin-converting enzyme 2 (ACE2) receptor and GRP78 receptor mediate severe acute respiratory syndrome coronavirus 2 (SARS-CoV-2) binding and internalisation in endothelial cells, leading to endothelial damage. Transmembrane protease serine 2 (TMPRSS2) mediate viral spike protein priming. The inhibition of GRP78 by monoclonal antibodies (hmAb159) reduces the viral binding to endothelial cells during COVID-19 infection. (**B**) The increased surface expression of GRP78 in endothelial and epithelial cells is seen in patients with diabetes mellitus, iron excess conditions and after corticosteroid therapy. Increased GRP78 expression may predispose COVID-19 patients to mucormycosis by enhancing the binding of Mucorales spores to the GRP78 receptor.

Endothelial cell dysfunction plays an important role in the pathogenesis of COVID-19 infections [30,33]. Severe acute respiratory syndrome coronavirus 2 (SARS-CoV-2) binds to the Angiotensin-Converting Enzyme 2 (ACE2) receptor on endothelial cells, followed by the internalisation of viral particles leading to coagulation, endotheliitis and endothelial cell death [33,34]. Other than the ACE2 receptor, Glucose Regulated Protein 78 (GRP78) acts as a co-receptor for the recognition of SARS-CoV-2 spike protein and increases the internalisation of the virus (Figure 1A) [31,35]. GRP78 is a heat shock protein with a molecular weight of 78 kDa, also known as Heat Shock Protein A5 (HSPA5). GRP78 is a molecular chaperone localised in the endoplasmic reticulum (ER) and has a significant role in regulating unfolded protein response (UPR), protein stability, calcium homeostasis and autophagy [36,37]. An increased serum GRP78 level has been noted in patients with COVID-19 infections compared to healthy controls [38]. Hence, the inhibition of the GRP78 receptor could reduce the internalisation of SARS-CoV-2 by reducing the expression of ACE2 receptors, further leading to reduced virus binding and infection severity [31,39]. Similar to the ACE2 receptor, GRP78 also mediates endothelial cell barrier disruption and inflammation [40].

Endothelial damage in COVID-19 is mediated by the activation of the inflammatory response, triggering an abnormal increase in pro-inflammatory cytokines (interleukin (IL) IL-1β, IL-6, IL-8, and tumour necrosis factor (TNF)-α), which alters the blood coagulation factors (D-dimer, von Willebrand factor (VWF), fibrinogen) leading to venous thrombosis, systemic vasculitis, endothelial cell apoptosis, vascular coagulopathy, and inflammation in various organs resulting in multi-organ failure [41,42,43]. The pre-existing endothelial damage in COVID-19 patients may act as an important risk factor for mucormycosis. Endothelial invasion is the critical step in the pathogenesis of mucormycosis [1]. The damaged endothelium and increased expression of endothelial receptors such as GRP78 in COVID-19 patients may increase the chance for angio-invasion and tissue necrosis.

Other than coronaviruses, GRP78 also acts as a receptor-binding site for Zika virus, Japanese Encephalitis virus, and Dengue virus [44]. Similar to SARS-CoV-2, the spike protein of Middle East respiratory syndrome coronavirus and bat coronavirus interacts with the GRP78 receptor and facilitates the attachment of the virus to the host cell and mediates the internalisation and replication of the virus [44,45]. Basal level GRP78 expression has been noted in healthy tissues, whereas increased expression of GRP78 has been reported in obesity, diabetes mellitus, metabolic syndrome, and cancerous tissues [36,37], which may predispose them to both COVID-19 infections and mucormycosis.

GRP78 receptors not only have a role in the pathogenesis of COVID-19 infections, but they are also crucial for mucormycosis pathogenesis, specifically in the ROCM type [46,47]. In the DKA mouse model of mucormycosis, the overexpression of the GRP78 receptor is seen in sinus, lung and brain tissues, and it is associated with increased adherence and endocytosis of *Rhizopus arrhizus* germlings to epithelial and endothelial cells [46,47]. CotH3 (spore coat protein) on the *R. arrhizus* cell surface acts explicitly as a fungal ligand for the GRP78 receptor [48]. The CotH3–GRP78 axis governs entry into the nasal epithelial and endothelial cells, explaining the increased number of ROCM cases in CAM. The genome analysis of Mucorales has revealed that CotH-like genes are conserved in this group of fungi [48,49]. CotH3 proteins act as invasins, and the binding of CotH3 proteins to GRP78 receptors mediates epithelial cell invasion and endothelial damage, leading to increased virulence [47,48]. Following binding to the endothelial cells, the spores to hyphae transition is mediated by the calcineurin pathway, which mediates angio-invasion [50,51]. The hyphal forms of *R. arrhizus* also produce mucoricin (ricin-like-toxin), which enhances angio-invasion, inflammation, and tissue destruction [52]. In COVID-19 patients, the increased expression of the GRP78 receptor may enhance the binding of Mucorales spores, resulting in enhanced endothelial invasion and damage (Figure 1B). The inhibition of the GRP78 receptor or the antibody against the CotH3 proteins can abolish the endothelial invasion by Mucorales spores [46,48]. Therefore, the role of GRP78 inhibitors in reducing the pathogenesis of COVID-19 infection and mucormycosis would be interesting to study.

Experimental data reported that the GRP78 receptor is not involved in the pathogenesis of pulmonary mucormycosis; Mucorales spores interact with the specific host cell type receptors depending on the site of infection [47]. In a murine model of pulmonary mucormycosis, CotH7 on the Mucorales spores interacts with the integrin β1 receptor on alveolar epithelial cells, thus inducing the activation of the epidermal growth factor receptor (EGFR), which mediates the fungal invasion of host cells [47,53]. Treatment with EGFR inhibitors (cetuximab or gefitinib) blocks the EGFR pathway, increasing the survival of mice with pulmonary mucormycosis [53]. Similarly, the inhibition of the integrin β1 receptor with anti-integrin β1 antibodies abolished the phosphorylation of EGFR and protected the neutropenic mice from pulmonary mucormycosis [47]. Thus, the CotH7–integrin β1–EGFR axis governs entry into airway epithelial cells, which plays a critical role in the pathogenesis of pulmonary mucormycosis. Further, in vitro interaction of Mucorales spores with endothelial cells showed the activation of platelet-derived growth factor receptor (PDGFR) signalling, which mediates Mucorales invasion of host cells, and the inhibition of PDGFR partially reduced host cell damage [49].

**Figure 2:**
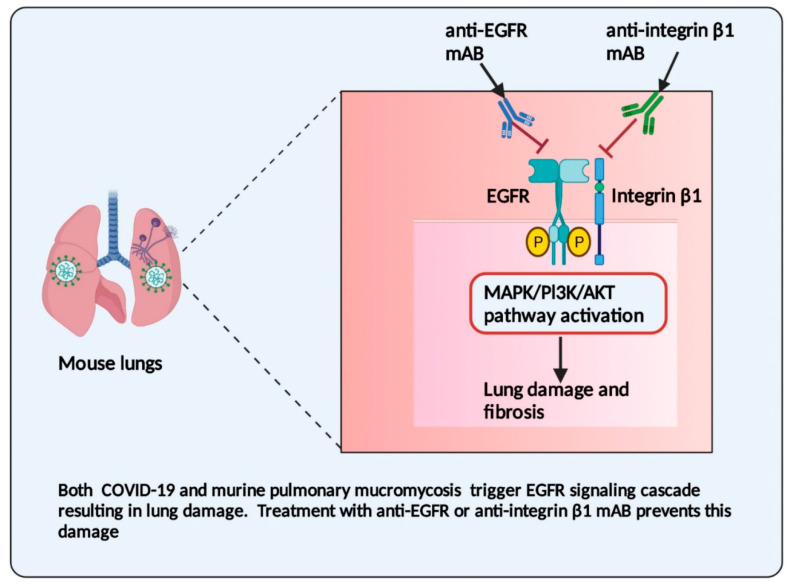
Epidermal growth factor receptor (EGFR) in the pathogenesis of COVID-19-associated mucormycosis. In COVID-19 disease and pulmonary mucormycosis, EGFR activation increases the lung pathology of both infections. EGFR activation is mediated by mitogen-activated protein kinase (MAPK), phosphoinositide 3-kinase (PI3K), and protein kinase B (AKT) pathways. Inhibition of EGFR by monoclonal antibody (mAB) or by drugs can reduce the lung damage and pathological features associated with COVID-19 and mucormycosis. The anti-integrin β1 antibody inhibits *Rhizopus*-induced phosphorylation of EGFR, leading to reduced lung damage in pulmonary mucormycosis.

Similarly, in the murine model of severe acute respiratory syndrome coronavirus (SARS-CoV) infection, the EGFR signalling pathway′s overexpression is associated with enhanced lung disease and lung fibrosis [54,55]. SARS-CoV-2 infection of colonic epithelial cells in vitro showed that the activation of growth factor receptors such as EGFR and PDGFR is associated with enhanced viral replication [32]. EGFR and PDGFR downstream signalling is activated by phosphoinositide 3-kinase (PI3K), protein kinase B (AKT) and mitogen-activated protein kinase (MAPK) pathways. The inhibition of these pathways by anticancer drugs reduced SARS-CoV-2 replication [32]. Like coronaviruses, other respiratory viruses such as influenza A virus and rhinovirus activate EGFR, leading to the suppression of antiviral defence mechanisms and increased viral infection [56,57]. Further, the inhibition of EGFR leads to reduced infection by influenza A and rhinovirus [56]. This experimental evidence indicates that the inhibition of EGFR signalling may prevent an excessive fibrotic response to coronavirus and other respiratory viral pathogens [57]. Interestingly, in pulmonary mucormycosis and COVID-19 infection, the activation of EGFR is necessary for their pathogenesis (Figure 2). The activated EGFR by COVID-19 may enhance the binding of Mucorales spores, thereby aggravating the pathogenesis of CAM.

### 3.2. Iron: The Possible Link between COVID-19 Infections and Mucormycosis

Hyperglycaemia is the major risk factor for mucormycosis. In patients with uncontrolled diabetes and poor glycaemic control, ketone concentration in the blood increases, which leads to acidosis—this condition is known as diabetic ketoacidosis (DKA) [46,58]. Iron binding proteins such as transferrin, ferritin and lactoferrin limit the release of free iron in the blood and maintain iron homeostasis [59]. In metabolic disorders such as DKA, the release of free iron in the blood increases, impairing iron homeostasis [58,59]. Patients with diabetes mellitus showed increased serum ferritin levels, leading to increased iron stores [27,60].

Similarly, COVID-19 interacts with the haemoglobin molecule and causes the dissociation of iron from heme molecules, which leads to hyperferritinaemia in COVID-19 patients [29,61]. Hyperferritinaemia alters iron homeostasis, leading to iron overload in the body [29,61]. Increased serum ferritin levels, IL-6 and D-dimer have been associated with high mortality in COVID-19 patients [62]. Hyperferritinaemia in COVID-19 patients is attributed primarily to inflammatory cells at the site of infection, such as macrophages, and increased pro-inflammatory cytokine secretion, such as IL-6. These factors contribute to increased lung inflammation and lung fibrosis, leading to severe disease [28,63].

Both hyperglycaemia and COVID-19 cause hyperferritinaemia, and the high ferritin inside the cells causes the release of oxygen-free radicals, which damages the tissue and releases free iron in the blood. The free iron in the blood favours Mucorales growth and invasion of blood vessels, causing vessel thrombosis and tissue necrosis [58,64]. Iron acquisition from the host is an important virulence attribute of Mucorales [64]. The genes that mediate iron acquisition by reductive pathways, such as the high-affinity iron permease (FTR1), multi-copper oxidase and ferric reductase, are conserved in the genomes of medically important Mucorales [65,66]. Mucorales also possess other iron acquisition genes such as siderophore transporters and heme oxygenase (uptake of iron from hemin) [65,66]. Further, Mucorales can store the acquired iron in the form of ferritins in their cells. Three types of ferritins reported in Mucorales are (a) mycoferritin, (b) bacterioferritin, and (c) zygoferritin [67].

The functional role of the high-affinity iron permease (*FTR*1) gene in the pathogenesis of mucormycosis has been demonstrated in *R. arrhizus* and *Lichtheimia corymbifera* [68,69]. The *FTR*1 genes are not up-regulated in iron-rich conditions; however, they play an important role in the growth and survival of Mucorales in iron-depleted conditions [68,69]. Increased expression of the *FTR*1 genes in *R. arrhizus* has been observed in DKA mice compared with the controls. *FTR*1 gene disruption (reduction in copy numbers) or the inhibition of gene expression by RNA interference reduces the iron acquisition capability of *R. arrhizus*, leading to decreased virulence in DKA mice [68]. The use of antibodies against the *FTR1* gene protected the DKA mice from mucormycosis [68].

Similar findings have been observed with *L. corymbifera* with in vitro experiments showing increased co-expression of multi-copper ferroxidase (*FET3*) and *FTR1* genes in *L. corymbifera* during iron-depleted conditions [69]. In *Mucor circinelloides*, the role of multicopper oxidase (fet3a, fet3b, fet3c) in the pathogenesis of mucormycosis has been elucidated [70]. The expression of the multicopper oxidase (fet3a, fet3b, fet3c) gene is strongly induced during iron-limiting conditions, suggesting their role in iron acquisition and growth. Of those, the fet3c gene plays a predominant role in the virulence of *M. circinelloides*. The fet3c mutant has demonstrated reduced virulence and mortality [70]. Similarly, in COVID-19 patients, Mucorales may use their iron acquisition genes and establish infection in a susceptible host because of the increased ferritin levels.

Iron chelating therapies have been used in medical practice to reduce iron overload in DKA patients, haemodialysis, renal failure, and transfusion-related disorders [71]. In COVID-19 infections, hyperferritinaemia is associated with vascular coagulopathy and endothelial inflammation, leading to multi-organ failure [72,73]. During COVID-19 infection, iron chelation therapies have been proposed to treat hyperferritinaemia [72,73]. However, iron-chelating agents such as deferoxamine predispose patients to mucormycosis [74,75]. In deferoxamine therapy, the siderophores of Mucorales can acquire iron from ferrioxamine (iron-deferoxamine complex) [76]. In vivo experiments on a murine model treated with deferoxamine showed increased iron uptake, germination and virulence by *R. arrhizus* [77,78]. The *Drosophila melanogaster* model of mucormycosis showed that deferoxamine-fed flies infected with *R. arrhizus* developed severe disseminated infections with a higher mortality rate than infected control flies with no deferoxamine [79]. It is observed that the Fob1/Fob2 proteins on the cell surface of *R. arrhizus* and other Mucorales spores act as a receptor to bind ferrioxamine and acquire iron from ferrioxamine by the reductase/permease-dependent pathway [78]. The Fob1/Fob2 and FTR1 mutant strain of *R. arrhizus* showed impaired iron uptake, germination, and reduced virulence in deferoxamine-treated mice [78].

Other iron chelators, such as deferasirox and deferiprone, showed reduced fungal burden in tissues and increased the survival rate in DKA and neutropenic mouse models of mucormycosis [77,80]. However, the siderophores on Mucorales cannot utilise iron from deferasirox and deferiprone [77,80]. Further, iron deprivation in in vitro conditions showed apoptosis of the *R. arrhizus* spores. These findings suggested that iron deprivation plays an important role in the pathogenesis of mucormycosis [81]. However, one must be cautious about using an appropriate iron chelator molecule to treat hyperferritinaemia in COVID-19 patients till clinical trials prove the actual utility.

### 3.3. Corticosteroid Therapy: A Friend-Turned-Foe in CAM

The World Health Organisation (WHO) strongly recommends using systemic corticosteroid therapy (6 mg of dexamethasone orally/intravenously daily or 50 mg of hydrocortisone intravenously every 8 h) for 7 to 10 days in patients with severe and critical COVID-19 infections. Further, they conditionally recommend not to use corticosteroid therapy in patients with non-severe COVID-19 and without any oxygen desaturation [82]. Glucocorticoids are widely used for their anti-inflammatory and immunosuppressive properties [83]. However, prolonged steroid use affects the immune system and leads to severe side effects such as hypertension, diabetes and osteoporosis [83]. Hyperglycaemia is the most common complication after glucocorticoid treatment [22]. Similarly, in previously diagnosed diabetic patients, the use of glucocorticoids is restricted due to the risk of disrupting glucose metabolism and the possible chances of acquiring insulin resistance [22,84]. In this group of patients, prolonged and inappropriate doses of steroid use can lead to severe immune suppression and the alteration of blood glucose levels [21,22]. Inappropriate steroid use is a significant risk factor for CAM cases [6,9,11].

The use of corticosteroid therapy suppresses the function of immune cells such as macrophages, neutrophils, platelets and T cells [26]. In anti-Mucor immunity, neutrophils and macrophages play a cardinal role, and the glucocorticoid-treated cells are defective in adherence, chemotaxis, oxidative burst and nitric oxide production [26,85]. Thus, the innate immune defects in phagocytic cells after glucocorticoid treatment make individuals highly susceptible to mucormycosis. Further, the experimental evidence reported that glucocorticoid treatment increases the surface expression of the GRP78 receptor [86,87]. Thus, the early use of dexamethasone in COVID-19 patients may increase GRP78 levels, leading to severe infections by increasing viral attachment and replication [88]. Similarly, glucocorticoid-induced immune defects and altered receptor expression may enhance the pathogenesis of CAM.

Multiple experimental studies have evaluated the innate response to Mucorales spores in healthy hosts and diabetes or glucocorticoid-induced immunocompromised conditions [79,85,89,90,91]. Alveolar macrophages from healthy mice failed to kill the *R. arrhizus* spores, though spore germination was suppressed [85,89,90]. In the *Drosophila melanogaster* model of mucormycosis, the phagocytosis of *R. arrhizus* spores was delayed under normal immune conditions [79]. In the Zebrafish model of mucormycosis, macrophages and neutrophils were actively recruited on infection with Mucorales spores. However, neutrophils alone played a protective role against Mucorales spores, whereas macrophages underwent apoptosis after interaction with Mucorales spores [92]. These experimental findings reported the reduced phagocytic potential of innate immune cells in the immunocompetent host.

**Figure 3:**
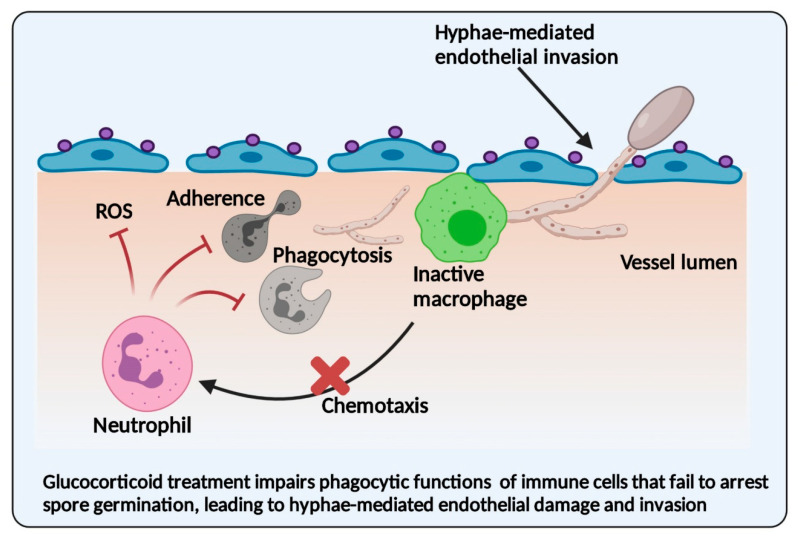
Corticosteroid therapy-induced immune dysfunction in COVID-19-associated mucormycosis. Corticosteroid therapy and diabetes mellitus attenuate the phagocytic functions of immune cells that allow the germination of Mucorales spores, leading to angio-invasion and tissue necrosis. Corticosteroid treatment impairs adherence, chemotaxis, phagocytosis and reactive oxygen species (ROS) production, failing to arrest spore germination and leading to hyphae-mediated endothelial invasion and damage.

In contrast to immunocompetent hosts, mice treated with cyclophosphamide or cortisone acetate showed severe infection and a higher mortality rate on infection with Mucorales [93]. Cortisone acetate treatment abolishes the phagocytic functions of macrophages, and they fail to inhibit the spore germination and killing of *R. arrhizus* spores [89]. Similarly, dexamethasone-treated *D. melanogaster* infected with *R. arrhizus* spores showed impaired phagocytic activity and reduced hyphal damage compared to immunocompetent flies [79]. During immunosuppression, the phagocytic potential of macrophages is compromised, and they fail to arrest the growth of Mucorales spores, which leads to hyphal invasion and progressive disease [79,89]. Dexamethasone-treated zebrafish infected with Mucorales spores showed reduced recruitment of macrophages and neutrophils to infection sites, resulting in higher mortality [94]. Like other immunosuppressed conditions, diabetes mellitus causes a delay in phagocytosis and killing inside the macrophages [25,89,90]. In patients with DKA, the impaired killing of Mucorales spores by neutrophils has been observed compared to healthy hosts [95]. These findings suggest that steroid treatment and diabetes mellitus reduce the phagocytic functions of macrophages and neutrophils against Mucorales spores. Similar to the experimental conditions, COVID-19 patients have impaired immune functions due to glucocorticoid therapy or diabetes mellitus, which predisposes them to CAM (Figure 3).

## 4. Conclusions

CAM outbreak is an important challenge during the COVID-19 pandemic in India. The emergence of mucormycosis in COVID-19 patients is attributed to environmental, host, and iatrogenic factors. The role of environmental factors needs to be evaluated to find the reason behind the unprecedented surge of CAM cases in India. The other possible reason for the surge could be uncontrolled diabetes and the consequence of COVID-19 itself. Pre-existing endothelial damage due to SARS-CoV-2 infection could trigger the pathogenic potential of Mucorales. Epithelial cell interaction and endothelial invasion mediate the pathogenesis of mucormycosis, and the endothelial breach in COVID-19 can increase the binding of Mucorales spores and initiate tissue invasion in the host. The over-expression of host receptors such as GRP78 and EGFR is associated with the pathogenesis of COVID-19 infections as they are essential for viral binding, internalisation and viral replication. Surprisingly, the GRP78 receptor on nasal epithelial cells and endothelial cells and integrin β1 and EGFR receptors on airway epithelial cells are essential for cell adhesion and tissue invasion by Mucorales. CotH proteins on Mucorales spores interact with different host receptors depending on the site of infection (CotH3–GRP78 in the ROCM type and CotH7–integrin β1–EGFR in pulmonary mucormycosis), which leads to disease progression.

Iron acquisition from the host is an important virulence trait of Mucorales. Patients with hyperglycaemia and COVID-19-induced inflammation often present with an increase in free iron in the blood, which predisposes them to mucormycosis. Mucorales use free iron by reductive iron acquisition pathway genes such as FTR1 and multicopper oxidase. The use of iron chelating agents such as deferoxamine to treat hyperferritinaemia in COVID-19 infections may also predispose them to mucormycosis. Mucorales spores bind to deferoxamine using their Fob1/Fob2 proteins on the cell surface, acquire iron from deferoxamine by the reductase/permease pathway, and initiate the pathological process. Further, in COVID-19 patients, inappropriately high doses of steroid use predispose them to mucormycosis. The prolonged use of steroids or an inappropriate dose of steroids suppresses the immune system, leading to complications such as steroid-induced diabetes. In addition, macrophage and neutrophil functions are impaired after steroid treatment with an attenuated oxidative burst and failure to kill the invading fungal spores, leading to disease progression. These overlapping pathogenic determinants between COVID-19 infections and mucormycosis may be the possible reason for the emergence of CAM.

However, further detailed studies are warranted to understand the pathogenesis of CAM. Future studies should be directed on: (a) the possible link between the immunosuppression mediated by the Delta or Delta plus variant of SARS-CoV-2 and their effect on Mucorales’ specific immunity; (b) the genetic susceptibility of the Indian population to mucormycosis; (c) the difference in virulence properties of Mucorales strains isolated during the CAM outbreak and those isolated before the COVID-19 pandemic; and (d) the other possible environmental and iatrogenic factors associated with the outbreak of CAM.
